# Histopathology of Cerebral Microinfarcts and Microbleeds in Spontaneous Intracerebral Hemorrhage

**DOI:** 10.1007/s12975-022-01016-5

**Published:** 2022-04-06

**Authors:** Wilmar M. T. Jolink, Susanne J. van Veluw, Jaco J. M. Zwanenburg, Annemieke J. M. Rozemuller, Wim van Hecke, Matthew P. Frosch, Brian J. Bacskai, Gabriël J. E. Rinkel, Steven M. Greenberg, Catharina J. M. Klijn

**Affiliations:** 1 Department of Neurology and Neurosurgery, University Medical Center Utrecht Brain Center, Utrecht University, G03.129, PO Box 85500, 3508 GA Utrecht, The Netherlands; 2 Department of Neurology, Isala Hospital, Zwolle, The Netherlands; 3 Department of Neurology, J. Philip Kistler Stroke Research Center, Massachusetts General Hospital and Harvard Medical School, Boston, MA USA; 4 Alzheimer Research Unit, Department of Neurology, Massachusetts General Hospital and Harvard Medical School, Boston, MA USA; 5 Department of Radiology, University Medical Center Utrecht, Utrecht, The Netherlands; 6 Department of Pathology, Amsterdam University Medical Centers, Amsterdam, The Netherlands; 7 Department of Pathology, University Medical Center Utrecht, Utrecht, The Netherlands; 8 Neuropathology Service, C.S. Kubik Laboratory for Neuropathology, Massachusetts General Hospital and Harvard Medical School, Boston, MA USA; 9 Department of Neurology, Donders Institute for Brain,Cognition and Behaviour, Centre for Neuroscience, Radboud University Medical Center, Nijmegen, The Netherlands

**Keywords:** Spontaneous intracerebral hemorrhage, Ultra-high-field MRI, Histopathology, Cerebral amyloid angiopathy

## Abstract

In patients with spontaneous intracerebral hemorrhage caused by different vasculopathies, cerebral microinfarcts have the same aspect on MRI and the same applies to cerebral microbleeds. It is unclear what pathological changes underlie these cerebral microinfarcts and cerebral microbleeds. In the current study, we explored the histopathological substrate of these lesions by investigating the brain tissue of 20 patients (median age at death 77 years) who died from ICH (9 lobar, 11 non-lobar) with a combination of post-mortem 7-T MRI and histopathological analysis. We identified 132 CMIs and 204 CMBs in 15 patients on MRI, with higher numbers of CMIs in lobar ICH patients and similar numbers of CMBs. On histopathology, CMIs and CMBs were in lobar ICH more often located in the superficial than in the deep layers of the cortex, and in non-lobar ICH more often in the deeper layers. We found a tendency towards more severe CAA scores in lobar ICH patients. Other histopathological characteristics were comparable between lobar and non-lobar ICH patients. Although CMIs and CMBs were found in different segments of the cortex in lobar ICH compared to non-lobar ICH patients, otherwise similar histopathological features of cortical CMIs and CMBs distant from the ICH suggest shared pathophysiological mechanisms in lobar and non-lobar ICH caused by different vasculopathies.

**Supplementary Information:**

The online version contains supplementary material available at 10.1007/s12975-022-01016-5.

## Introduction

The etiology of spontaneous intracerebral hemorrhage (ICH) is still poorly understood. Classically, non-lobar (deep and infratentorial) ICH has been associated with arteriolosclerotic vasculopathy of the deep penetrating blood vessels caused by longstanding high blood pressure, diabetes and alcohol overuse [1, 2], and lobar ICH in elderly patients with cerebral amyloid angiopathy (CAA) in the leptomeningeal and cortical blood vessels [3]. However, evidence is accumulating that arteriolosclerotic vasculopathy and CAA often exist together, and clinical and imaging phenotypes may overlap [4, 5]. High blood pressure is not only an important risk factor for non-lobar ICH, but also for lobar ICH, although with a smaller effect [2]. On MRI, lobar and non-lobar ICH share manifestations of cerebral small vessel disease, including white matter hyperintensities, enlarged perivascular spaces, lobar and deep cerebral microbleeds (CMBs), and cortical superficial siderosis [3, 6–11]. Several studies have also identified focal ischemic lesions in patients with lobar and in patients with non-lobar ICH [12, 13]. Small diffusion-weighted imaging-positive lesions, indicative of acute cerebral microinfarcts (CMIs), are found in both lobar and deep regions of the brain in approximately 20% of patients with lobar or non-lobar ICH [13, 14]. With high field MRI, chronic CMIs can be visualized in the cortex [15, 16]. This method recently revealed that chronic cortical CMIs are common in patients with lobar and in those with non-lobar ICH and that they co-occur with (deep and lobar) CMBs [17]. In patients with spontaneous ICH caused by different vasculopathies, CMIs in different locations have the same aspect on MRI and the same applies to CMBs. It is unclear what pathological changes underlie these MRI-visible CMIs and CMBs. We hypothesized that histopathological characteristics are different according to the underlying vasculopathy. The aim of this study was to determine whether the histopathological characteristics and their location in the cortex of CMIs and CMBs found on ex vivo high-resolution 7 tesla (T) MRI scans differ in terms of presence and severity of CAA, arteriolosclerosis, concentric vessel wall splitting, and loss of smooth muscle cells and presence of fibrin(ogen) in the vessels in close proximity of the lesions and the surrounding area between patients with lobar and non-lobar ICH.

## Methods

### Patients

From the database of autopsy cases of the University Medical Center Utrecht (UMCU), we included 12 consecutive patients who died as a consequence of a spontaneous ICH (not including traumatic ICH or when a secondary cause was found such as a macrovascular cause or malignancy) and underwent autopsy between 2008 and 2015. One of the included patients had hereditary Dutch-CAA (D-CAA; also known as hereditary cerebral hemorrhage with amyloidosis – Dutch type, a hereditary form of CAA), which had been diagnosed during life. We selected eight additional consecutive patients from the Netherlands Brain Bank of patients who died because of spontaneous ICH between 1997 and 2013. The presumed cause (i.e., CAA or arteriolosclerotic vasculopathy) of the ICH was extracted from the pathology reports from routine pathological examination, which included screening for arteriolosclerotic changes and Congo red stain and/or immunohistochemistry against amyloid β (Aβ). Hypertension was defined as a reported history of hypertension or use of antihypertensive medication (not only in the acute phase of the ICH) as registered in the pathology reports and/or the medical record for the patients from the UMCU. We determined presence of large vessel atherosclerosis based on the information in the pathology reports.

The brain samples obtained from the Netherlands Brain Bank, Netherlands Institute for Neuroscience, Amsterdam (open access: www.brainbank.nl), had been collected from donors that had provided written informed consent for the use of autopsy material and clinical information for research purposes. For patients from the UMCU, informed consent was obtained prior to autopsy, according to local ethical guidelines. The study was approved by the medical research ethics committee of the UMCU.

### MRI Acquisition and Analysis

From each patient, we selected three 10-mm-thick formalin-fixed coronal brain slabs from the frontal, parieto-temporal (ideally including part of the basal ganglia), and occipital regions of the available brain tissue (in most cases only the hemisphere contralateral to the ICH). Per patient we submerged the three slabs in 10% formalin in a purpose-built Perspex container that fitted in the head coil of the MR scanner. We specifically removed any air bubbles, because gradient echo sequences are susceptible to artifacts caused by air bubbles. Scans were acquired on a whole body 7-T MRI system (Philips, Best, the Netherlands) with a dual transmit and 32-channel receive head coil (Nova Medical, USA). We scanned the post-mortem brain tissue overnight for approximately 14 h with a protocol including a 3D fluid attenuated inversion recovery sequence, a 3D T_2_-weighted turbo spin echo, a 3D T_1_-weighted, and a 3D T_2_*-weighted sequence. A more detailed scan protocol is available from the online data supplement.

The acquired MR images were rated for cortical CMIs and CMBs by two readers (WMTJ, SJvV) independently and blinded to hemorrhage location and diagnosis. CMIs were defined as small (≤ 5 mm) cortical lesions, hyperintense on T_2_, isointense on T_2_* and hypointense on T_1_-weighted sequences.^10, 11^ We only looked at cortical CMIs, since the current detection criteria do not discriminate CMIs in deeper areas of the brain from other pathologies, such as enlarged perivascular spaces and white matter hyperintensities [18]. CMBs were defined as small (≤ 10 mm) round or ovoid lesions of signal void on T_2_-weighted sequences with associated blooming on T_2_*-weighted sequence [19, 20]. For CMBs, we screened for both cortical and deep CMBs (in basal ganglia and thalamus). Lesions were annotated in MeVisLab (MeVis Medical Solutions, Bremen, Germany). We determined the location of the CMIs and CMBs in the cortex as superficial (touching the outer border of the cortex; predominantly in the upper half of the cortex) or deep (not touching the cortex; predominantly in the lower half of the cortex).

### Tissue Sampling, Histopathological, and Microscopic Analysis

From each patient with lesions identified on 7-T MRI, we took at least three samples, with, if present, multiple lesions per sample and at least one CMI and CMB. In addition, from the D-CAA patient, we took a total of eight samples to allow a more extensive comparison of this patient with the sporadic CAA patients.

All samples were dehydrated, embedded in paraffin, and cut in 6-µm-thick sections on a microtome. Guided by the MR images, we attempted to retrieve the lesions; based on tissue architecture, we estimated the depth of the lesion in the tissue block. We took ten sections around the estimated lesion location at three depths with a slice gap of approximately 500 μm. Standard hematoxylin & eosin (H&E) staining was performed on the first sections of the three series. After successful retrieval of lesions, adjacent sections underwent Perls’ Prussian Blue staining (for iron) and immunohistochemistry against Aβ (clone 6F/3D, Agilent, Dako), smooth muscle cells (SMC; Agilent, Dako), and fibrin(ogen) (Agilent, Dako), using methods that have previously been described in detail [21].

All sections were imaged with brightfield microscopy using the Hamamatsu NanoZoomer Digital Pathology-HT scanner (C9600-12, Hamamatsu Photonics KK, Japan) and examined by two observers (WMTJ and SJvV) blinded for hemorrhage location and diagnosis, using the viewing platform NDP.View (version 2.6.13.0). If needed, sections were discussed with two experienced neuropathologists (AJMR, MPF). CMIs and CMBs were evaluated on H&E-stained sections. CMI identification was based on areas of tissue pallor with evidence of eosinophilic necrosis or “red” neurons (acute CMIs) or cell loss with cavitation or “puckering” (chronic CMIs). CMBs were identified by the presence of erythrocyte extravasation (acute CMBs) or blood breakdown products, such as hematoidin or hemosiderin (chronic CMBs). Vessels near the lesions and on adjacent sections were examined to identify the involved vessel. We determined the location of the lesion in the cortex (superficial/along penetrating cortical arteriole [layers I–III], deeper in cortex [layers IV–VI] or subcortical). The severity of CAA was scored as described by Vonsattel [22–24] and presence of capillary CAA defined as type 1 (capillary CAA present) and type 2 (no capillary CAA) [22]. We evaluated presence of arteriolosclerosis on H&E [23, 24]. We scored presence of concentric vessel wall splitting (as a marker of severity of both CAA-related an arteriolosclerotic vasculopathy) [24–26] and loss of SMCs in the walls of vessels in close proximity to the lesions and for vessels present in the surrounding area (i.e., average impression of the whole section containing the lesion) of the CMI and CMB, using a semi-quantitative 4-point scale (0 = absent, 1 = mild, 2 = moderate, and 3 = severe) for each marker. In addition, we determined the presence of fibrin(ogen) in the walls of the involved vessel and in the surrounding cells around a lesion, also on a 4-point scale (0 = absent, 1 = mild, 2 = moderate, and 3 = severe), as a measure for blood–brain barrier leakage [21]. We used a prespecified assessment form to score characteristics of each identified lesion. The assessment form is provided in the online supplemental material.

### Statistical Analysis

We used the *χ*^2^ test, Fisher’s exact test, and Mann–Whitney *U* test, as appropriate, to analyze group differences in number of MRI-observed CMIs and CMBs, lesion location in the cortex, CAA severity (score ≥ 2 vs < 2), severity of vessel wall splitting (score ≥ 2 vs < 2), loss of SMCs (score ≥ 2 vs < 2), and presence of fibrin in vessel wall and surrounding cells (score ≥ 2 vs < 2) between patients with lobar and non-lobar (i.e., deep and infratentorial) ICH, and between sporadic CAA and D-CAA. We used multivariable logistic regression analysis to adjust all statistically significant (*p* < 0.05) variables for age at time of death and sex. For these descriptive analyses, we considered each lesion as an independent variable.

## Results

Table 1 summarizes the characteristics of the 20 included patients. Median age at time of death was 77 years (interquartile range [IQR] 51–83) and 55% were females. Location of the ICH was lobar in nine patients and non-lobar in 11 patients (eight deep and three infratentorial ICH). Median age at death in patients with lobar ICH was 78 years (IQR 75–82) and in non-lobar patients 55 years (IQR 45–85). One out of nine patients with lobar ICH and 6/11 of patients with non-lobar ICH had a known history of hypertension. Based on the routine pathological examination six of the nine patients with lobar ICH had sporadic CAA, one patient had D-CAA (genetically proven during life), one patient showed mixed pathology (abnormalities consistent with both arteriolosclerotic vasculopathy and CAA), and one patient showed neither arteriolosclerotic vasculopathy nor CAA. Of the 11 patients with non-lobar ICH, five had abnormalities consistent with arteriolosclerotic vasculopathy in the basal ganglia, two patients had mixed pathology (abnormalities indicating both arteriolosclerotic vasculopathy and CAA), and four patients had another cause (Table 1).

**Table 1:** Patient characteristics

Case no	Age at death (years)	Sex	Location ICH	Hemisphere	Hypertension	Diagnosis (based on PA reports)	Large vessel atherosclerosis^a^	No. of days between ICH onset and death
1	56	M	Lobar	Right	No	D-CAA^b^	Mild	10
2	76	M	Lobar	Right	No	CAA	Mild	2
3	78	F	Lobar	Left	No	Lobar ICH, no CAA	Mild to moderate	13
4	80	M	Lobar	Unknown	Unknown	CAA	Unknown	Unknown
5	85	M	Lobar	Right	No	CAA and arteriolosclerotic vasculopathy	Moderate to severe	4
6	78	M	Lobar	Both	Yes	CAA	None	5
7	83	F	Lobar	Right	No	CAA	Moderate	Unknown
8	81	F	Lobar	Left	No	CAA	Severe	1
9	73	F	Lobar	Left	No	CAA	Unknown	Unknown
10	81	M	Deep	Right	Yes	Arteriolosclerotic vasculopathy	Moderate to severe	Unknown
11	89	F	Deep	Left	Yes	Arteriolosclerotic vasculopathy	Moderate to severe	Unknown
12	89	F	Deep	Left	Yes	Arteriolosclerotic vasculopathy and CAA	Severe	Unknown
13	49	F	Deep	Left	Yes	Arteriolosclerotic vasculopathy	Mild to moderate	3
14	44	F	Deep	Left	No	Arteriolosclerotic vasculopathy	Mild to moderate	1
15	46	M	Deep	Right	Yes	Arteriolosclerotic vasculopathy	Severe	1
16	40	F	Deep	Right	No	Coagulation disorder in hepatic insufficiency	Mild	1
17	85	F	Deep	Right	Yes	Arteriolosclerotic vasculopathy and CAA	Severe	Unknown
18	55	M	Cerebellum	Left	No	Minimal arteriolosclerotic vasculopathy	Moderate	1
19	75	F	Pons	NA	No	Arteriolosclerotic vasculopathy and use of oral anticoagulants	Severe	1
20	45	M	Pons	NA	No	Atherosclerosis large vessels, lacunae, no other abnormalities small vessels	Moderate	1

*CAA* cerebral amyloid angiopathy, *D-CAA* Dutch-type CAA, *F* female, *ICH* intracerebral hemorrhage, *M* male; ^a^Based on PA report; ^b^Diagnosed during life by genetic testing

### Ex Vivo* 7-T MRI Findings*

On the ex vivo 7-T MR images, four patients had no microvascular lesions, including one patient with CAA (no. 6), one patient with deep ICH associated with a coagulation disorder in hepatic insufficiency (no. 16), one patient with cerebellar ICH (no. 18), and one patient with a pons hemorrhage (no. 15).

**Fig. 1:**
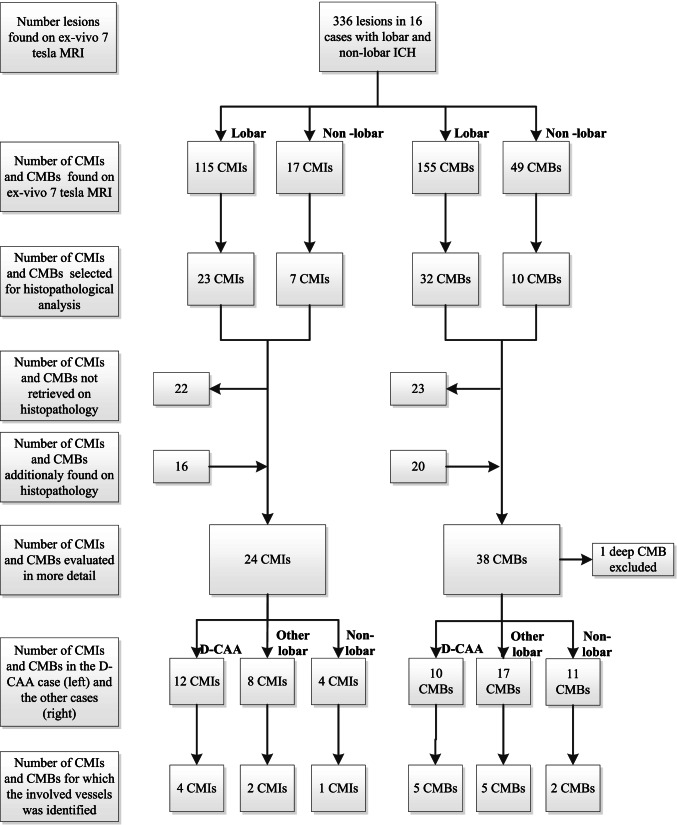
Flowchart of identified, retrieved, and evaluated lesions. CMB, cerebral microbleed; CMI, cerebral microinfarct; D-CAA, Dutch-type hereditary CAA

In the patient with D-CAA, we found a total of seven CMIs and 29 CMBs (all cortical) on ex vivo 7-T MRI, which we analyzed separately as described below (see Fig. 1 for a flowchart of the identified, retrieved, and evaluated CMIs and CMBs).

In the remaining 15 patients, we identified a total of 125 cortical CMIs, 170 cortical CMBs, and 5 deep CMBs on ex vivo 7-T MRI. In patients with lobar ICH (*n* = 7; six with CAA) compared to non-lobar ICH (*n* = 8; two with mixed pathology), we found more CMIs (lobar ICH: median 6 (IQR 1–33), non-lobar ICH: median 1 (IQR 0–2), *p* = 0.03). We also found a higher total number of CMBs in lobar ICH patients, but the median number per patient was not significantly different between lobar (median 1 (IQR 0–42)) and non-lobar ICH (median 2 (IQR 0–8)) patients (*p* = 0.83). After evaluation of the pathology slides, we excluded one patient with lobar ICH and CAA (no. 9) from further analysis, because of the presence of extensive hypoxic-ischemic changes throughout the tissue. Notably, the exclusion of this patient did not change the finding of more CMIs on MRI in the lobar ICH patients (median 3, IQR 0–54), compared to the non-lobar ICH patients (median 1, IQR 0–2, *p* = 0.05).

**Fig. 2:**
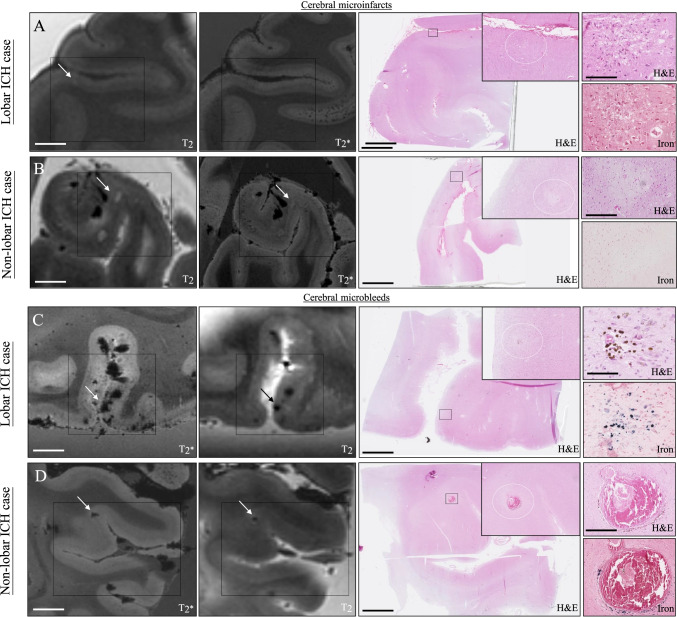
Representative examples of the different cortical location of CMIs and CMBs on ex vivo 7-T MRI and the matched histopathological sections in lobar and non-lobar ICH patients. In this figure, we show representative examples of cerebral microinfarcts (**A**, **B**) and microbleeds (**C**, **D**) in patients with lobar ICH (**A** [case no. 1] and **C** [case no. 8]) and non-lobar ICH (**B** [case no. 17] and **D** [case no. 11]) indicated with the black and white arrows. From each lesion, we show how we matched the lesions found on ex vivo 7 T T_2_ and T_2_*-weighted images with the histological section on an overview of the H&E section and the identified lesion on H&E and iron staining. The insert in the overview of the H&E section shows in more detail the location of the CMIs and CMBs in the superficial layers of the cortex in lobar ICH (**A**, **C**) and in the deeper layers of the cortex in non-lobar ICH (**B**, **D**). Scale bars in the T_2_ and T_2_*-weighted images are 10 mm; in the overview of the H&E section, 5 mm; in the more detailed H&E and iron staining sections of panels **A** and **C**, 200 µm; and the H&E and iron staining sections of panels **B** and **D**, 400 µm

We selected 71 of the lesions that we identified on MRI (30 CMIs and 41 CMBs) for histopathological analysis. On the corresponding sections, we could retrieve 27 lesions (38%; 20% of the CMIs and 51% of the CMBs). The lesions we could not retrieve were mostly small. We found 35 additional lesions after screening each section included in the MRI-targeted analysis, of which 18 could be identified on MRI in retrospect. Histopathology of the targeted MRI lesions confirmed the lesions as CMI or CMB in all (Fig. 2). In three patients (1 lobar [no. 6] and two deep ICH [no. 10 and 14]), we could not retrieve the targeted lesions and found no additional lesions. We could also not retrieve the targeted lesions in deep areas (case no. 15); hence, we limited the analysis to the 62 cortical lesions, 40 lesions in 10 patients with lobar or non-lobar ICH, and 22 lesions in the patient with D-CAA.

### Histopathological Characteristics of Areas Around CMBs and CMIs in Lobar and Non-lobar ICH Patients

In patients with lobar ICH, the cortical CMIs and CMBs were on histopathology more frequently located in the superficial layers of the cortex (layers I–III; lobar ICH: 63% of the CMIs and 71% of the CMBs, non-lobar ICH 9% of the CMBs and 25% of the CMIs; *p* = 0.001), whereas in non-lobar ICH patients, CMIs and CMBs were mostly located in the deeper layers of the cortex (layers IV–VI; lobar ICH: 38% of the CMIs and 6% of the CMBs, non-lobar ICH: 50% of the CMIs and 55% of the CMBs; *p* = 0.03; Table 2 and Fig. 2). In a multivariable model, correcting for age and sex superficial cortical location of CMIs and CMBs remained associated with lobar ICH (*p* = 0.002) and deeper cortical location of CMIs and CMBs with non-lobar ICH (*p* = 0.04). When we evaluated the corresponding CMIs and CMBs on the 7-T MR images in retrospect, differences in cortical lesion location between patients were not significant (*p* < 0.20). This was due to five lesions (3 CMIs and 2 CMBs) that were not identified on MRI in retrospect and four lesions (1 CMI and 3 CMBs) that were classified as located in the deep cortical layers on the section and superficial on MRI.

**Table 2:** Histopathological characteristics of identified lesions in lobar and non-lobar ICH

Lobar vs non-lobar ICH	Lobar ICH* (*n* = 25 lesions)†	Non-lobar ICH (*n* = 15 lesions)‡	*p*-value
Type of lesion§			
- CMI, *n* (%)	8 (32)	4 (26.7)	
- CMB, *n* (%)	17 (68)	11 (73.3)	
Location of lesion			
- Occipital, *n* (%)	6 (24)	5 (33.3)	
- Parieto-temporal, *n* (%)	13 (52)	7 (46.7)	
- Frontal, *n* (%)	6 (24)	3 (20)	
Location in the cortex			
- Superficial cortex (layers I–III)	17 (68)	2 (13.3)	0.001
- Deep cortex (layers IV–VI)	4 (16)	8 (53.3)	0.03
- Subcortical	4 (16)	5 (33.3)	0.26
Presence of microaneurysms, *n* (%)	0 (0)	1 (6.7)	0.38
Presence of fibrinoid necrosis, *n* (%)	8 (32)	4 (26.7)	0.72
CAA score ≥ 2	19 (76)	7 (46.7)	0.09
VWS score ≥ 2	19 (76)	8 (53.3)	0.18
Loss of SMCs score ≥ 2 ||	14/21 (56)	8/14 (57.1)	0.72
Fibrin leakage score ≥ 2			
- Vessel walls#	5/10 (50)	4/10 (40)	0.65
- Surrounding cells**	10/19 (52.6)	11/15 (73.3)	0.30

*CAA* cerebral amyloid angiopathy, *CMB* cerebral microbleed, *CMI* cerebral microinfarct, *ICH* intracerebral hemorrhage, *SMC* smooth muscle cell, *VWS* vessel wall splitting

^*^Excluding the patient with D-CAA

^†^Cases: no.2 (2 CMIs, 2 CMBs), no 3. (1 CMI, 3 CMBs), no. 7 (4 CMBs), no. 8 (1 CMI, 8 CMBs), no. 9(4 CMIs)

^‡^Cases: no. no 11. (4 CMBs), no. 12 (2 CMBs), no. 13 (2 CMIs), 17 (2 CMIs, 4 CMBs), no. 19 (CMB)

^§^We performed no statistical test for this variable, because it is a selection of lesions based on MRI

||In 5 lesions (4 in lobar ICH patients and 1 a non-lobar ICH patient), the vascular smooth muscle cell-stained section was not available

^#^In 14 lesions (9 in lobar ICH patients and 5 in non-lobar ICH patients), the involved vessels were not identified and in 6 lesions (all in lobar ICH patients), no fibrin(ogen)-stained section was available

^**^In 6 lesions (all in lobar ICH patients), the fibrin(ogen)-stained section was not available

Patients with lobar ICH tended to have more often moderate or severe CAA (score ≥ 2) in the surrounding areas of CMIs and CMBs compared to non-lobar ICH patients (*p* = 0.09; supplemental Fig. I and Table 2). Lobar and non-lobar ICH patients were otherwise comparable in terms of presence of moderate or severe vessel wall splitting (score ≥ 2) in the surrounding area (*p* = 0.18), moderate or severe loss of SMCs (score ≥ 2) (*p* = 0.72), and moderate or severe leakage of fibrin (score ≥ 2) in the surrounding vessel walls (*p* = 0.64) and the surrounding cells (*p* = 0.30).

### Exploratory Comparisons Between Sporadic CAA and D-CAA

In the patient with D-CAA (age at death 56 years), we found a total of 22 lesions with the combined MRI-targeted and -untargeted approach. Twelve of the 22 lesions were CMIs and ten were CMBs. We compared these lesions to 21 lesions found in four patients with lobar ICH and sporadic CAA (median age at death 80, IQR 75–82), including seven CMIs and 14 CMBs,

The location of the lesions in the cortex was in the superficial layers (layers I–III) in the majority of lesions in sporadic CAA patients (61.9%) and the D-CAA patient (77.3%; supplemental Table I). Presence of moderate or severe CAA (score ≥ 2) in the surrounding areas in sporadic CAA patients (90.5% of lesions) was comparable to the D-CAA patient (100%). All patients with sporadic CAA had CAA type 2 (i.e., no capillary CAA) and the D-CAA patient had CAA type 1 (i.e., presence of capillary CAA). Between sporadic CAA and D-CAA, we also found similar percentages of other characteristics (see Supplemental Table I and supplemental Fig. II for representative examples).

### Descriptive Findings Regarding the Involved Vessels

From the 40 lesions in lobar and non-lobar ICH patients, we were able to identify the involved cortical vessel for three CMIs and seven CMBs, allowing only a qualitative assessment of these “culprit” vessels involved in CMBs. Notably, even though the vessel walls appeared abnormal (see, for example, Fig. 2, panel D), severe CAA (score ≥ 2) of the involved vessels was infrequently observed in both non-lobar (1 of 3 CMBs) and lobar ICH patients (0 of 4 CMBs) (see, for example, supplemental Fig. I, panel C). In comparison, we identified the involved vessel for five CMBs in the D-CAA patient. Interestingly, 4/5 vessels involved in CMBs showed severe CAA (see, for example, supplemental Fig. II, panel C).

## Discussion

This study found more CMIs, but a similar number of CMBs on 7-T MRI in lobar ICH patients compared to non-lobar ICH patients, while on histopathology, the underlying histopathological signature, in terms of presence and severity of CAA, arteriolosclerosis, concentric vessel wall splitting, and loss of smooth muscle cells and presence of fibrin(ogen) in the vessels in close proximity to the lesions and the surrounding areas of the CMIs and CMBs in the cortex, was comparable in lobar and non-lobar ICH. CMIs and CMBs in lobar ICH were located predominantly in the superficial layers of the cortex whereas in non-lobar ICH, these lesions occur mostly in the deeper layers of the cortex. We found a tendency towards more severe CAA scores in lobar ICH patients, but severity of vessel wall splitting, loss of smooth muscle cells, and fibrin leakage around CMIs and CMBs was similar in lobar and non-lobar ICH with underlying sporadic CAA, arteriolosclerosis, and D-CAA etiologies.

The characteristic histopathological features of CAA include accumulation of Aβ in the media of parenchymal arterioles with progressive loss of smooth muscle cells and secondary changes consisting of fibrinoid necrosis, vessel wall thickening, microaneurysm formation, and perivascular deposition of blood break down products [3, 22, 26]. Arteriolosclerotic vasculopathy is characterized by changes in small, perforating vessels with collagenous vessel wall thickening with lumen narrowing and also progressive loss of smooth muscle cells, exudation of fibrin and other serum proteins, microaneurysms, fibrinoid necrosis, and lipohyalinosis [1, 3, 27, 28]. We found similar histopathologic characteristics of CMIs and CMBs in lobar and non-lobar ICH, which is in line with accumulating evidence suggesting that the histopathological features of the two types of cerebral small vessel disease often co-exist in patients [4]. A recent study found that 42% of participants with lobar ICH had moderate or severe arteriolosclerosis in addition to moderate or severe CAA on pathological examination [4]. Moreover, in that study, 39% of participants with lobar ICH had moderate or severe arteriolosclerosis alone and 13% of participants with non-lobar ICH had moderate or severe CAA [4]. Another study describing a neuropathological cohort of ICH patients showed that 11 of the 38 (28.9%) patients with hypertension and lobar or non-lobar ICH had CAA and that 61% of ICH patients with CAA had hypertension, suggesting an interaction between CAA and arteriolosclerotic vasculopathy in the pathophysiology of ICH [5]. However, this study did not report the presence of arteriolosclerotic changes in the vessels [5]. In contrast to traditional conceptualization, CAA and arteriolosclerotic vasculopathy may more commonly co-occur than previously thought and may share some pathophysiological pathways [5, 29, 30].

We found that in patients with lobar ICH, CMIs and CMBs were mainly located in the superficial layers of the cortex. This is in line with a recent study of 12 patients with definite CAA, which found more than 50% of the described CMIs (49%) and CMBs (77%) in the more superficial layers of the cortex [31]. We also found this percentage to be higher for CMBs (71%) than CMIs (63%). In our study, we were able to study patients with non-lobar ICH as well. We found that in non-lobar ICH patients, CMIs (55%) and CMBs (50%) were more often found in the deeper layers of the cortex. This finding suggests that different types of vasculopathy affect distinct categories of arterioles. Superficial perforating cortical arterioles are potentially more vulnerable to CAA and deep perforating cortical arterioles more to arteriolosclerosis, but the histopathological consequences of the impingement appear similar.

We also did an exploratory analysis of individual vessels involved in CMIs and CMBs. The abnormal appearance of vessels involved in CMBs in both lobar and non-lobar ICH patients in our study suggests reduced vessel integrity including vessel wall breakdown. In accordance with previous observations, the absence of severe CAA in these involved vessels suggests that the presence of vascular Aβ is not necessarily required to induce bleeding, but that complex pathways — potentially involving vascular remodeling — may play a role [31, 32]. Regarding CMIs, a shared underlying pathophysiology in lobar and non-lobar ICH may include thickening of the vessel wall with increased CAA severity (in lobar ICH), and loss of SMCs, although this notion requires further experimental investigations [31].

In lobar ICH compared with non-lobar ICH patients, but also in sporadic CAA patients compared with the D-CAA patient, we found in 40–70% of lesions blood–brain barrier leakage, expressed by the presence and severity of fibrin in the vessel walls and surrounding cells of CMIs and CMBs. This is in line with a recent systematic review of 10 animal studies and 16 studies in humans showing indications of blood–brain barrier dysfunction in both ICH related to CAA (9 of 12 studies) and arteriolosclerotic vasculopathy-related ICH (4 out of 5 studies) [33].

Despite the difference in age at death between the sporadic CAA patients (median age at death 80 years) and the D-CAA patient (age at death 56 years), overall CAA burden and individual histopathological features of CMIs and CMBs were comparable [34], except for the increased deposition of vascular Aβ in walls of vessels involved in CMBs in the D-CAA patient.

Strengths of our study are the combination of post-mortem 7-T MRI and histopathological examination to identify and characterize microvascular lesions in patients with spontaneous ICH. On routine autopsy, these lesions are often missed. Using 7-T MRI to target lesions for sampling, we were able to substantially increase the number of lesions available for detailed histopathological analysis.

Our study also had limitations. First, our sample size was relatively small. Nevertheless, for spontaneous ICH, in which autopsy is not performed routinely, it is one of the larger samples currently described. Second, of all lesions identified by MRI, we selected 71 of the 336 lesions for further analysis, based on the highest likelihood of successful retrieval of at least one CMI and one CMB and if possible multiple lesions per sample per slab. This approach may have introduced a selection bias. Unfortunately, we were only able to retrieve less than half of the lesions for histopathological analysis, likely due to the fact that we estimated lesion location based on MRI rather than performing serial sectioning through the entire samples, which was chosen as the approach for this project for practical reasons. For the same reason, we were not able to retrieve the “culprit” vessel for all lesions, limiting our analysis to a descriptive examination. Third, we only used a semi-quantitative (4-point) scale to score smooth muscle cells covering, limiting the information about the arteriolar vasculature in the region of the lesions. Fourth, patients in our study might not be representative of the whole spectrum of ICH, because autopsy is not routinely performed in patients who die of ICH, resulting in a heterogeneous study population Fifth, in our analyses, we considered each lesion as an independent variable, even when lesions were obtained from the same case. We did not account for co-dependence, because of the small number of lesions and exploratory nature of our study. Sixth, due to the retrospective nature of our study, we had some missing data concerning baseline characteristics. In addition, we did not have a CT or MRI of the acute ICH in all patients. Finally, although we screened for both cortical and deep CMBs, we retrieved only one deep CMB on microscopic examination.

## Conclusions

This exploratory study, using a lesion-targeted approach with a combination of 7-T MRI and histopathology, contributes to a better understanding of the cerebral small vessel disease underlying ICH. Further studies, preferably in a larger post-mortem cohort of ICH patients, including serial sectioning and if possible also investigating the perihematomal site are needed to confirm our findings. It would also be of interest to confirm in vivo our finding that CMIs and CMBs appear to occur in different layers of the cortex according to their presumed underlying vasculopathy.

Although CMIs and CMBs were found in different segments of the cortex in lobar ICH compared to non-lobar ICH patients, otherwise similar histopathological features suggest possible shared pathophysiological mechanisms in cerebral amyloid and arteriolosclerotic vasculopathies. Thickening of vessel walls, vascular remodeling, and blood–brain barrier dysfunction may be triggered by accumulation of amyloid or arteriolosclerotic vasculopathy, or both, and potentially cause CMIs and CMBs.

## Supplementary Information

Below is the link to the electronic supplementary material.

Supplementary file1 (DOCX 741 KB)

## Data Availability

The dataset analyzed in this study is not publicly available due to restricted access. Further information about the dataset is available from the corresponding author on reasonable request.
