# Supplementary material for: Histopathology of Cerebral Microinfarcts and Microbleeds in Spontaneous Intracerebral Hemorrhage
Source: Transl Stroke Res. 2022 Apr 6;14(2):174–84. doi: 10.1007/s12975-022-01016-5 (PMC9995541; doi:10.1007/s12975-022-01016-5)
Supplement: Supplementary file 1 — Supplementary file1 (DOCX 741 KB) [file 12975_2022_1016_MOESM1_ESM.docx]

SUPPLEMENTAL MATERIAL

Histopathology of cerebral microinfarcts and microbleeds in spontaneous intracerebral hemorrhage

*Translational Stroke Research*

*Manuscript ID #TRSR-D-21-00569*

Wilmar MT Jolink MD, Susanne J van Veluw PhD, Jaco JM Zwanenburg PhD, Annemieke JM Rozemuller MD PhD, Wim van Hecke MD PhD, Matthew P Frosch MD PhD, Brian J Bacskai PhD, Gabriël JE Rinkel MD PhD, Steven M Greenberg MD PhD, Catharina JM Klijn MD PhD

Corresponding author:

W.M.T. Jolink

Department of Neurology and Neurosurgery, University Medical Center Utrecht Brain Center, Utrecht University, Utrecht, The Netherlands and Department of Neurology, Isala Hospital, Zwolle, The Netherlands

E-mail: [w.m.t.jolink@isala.nl](mailto:w.m.t.jolink@isala.nl)

Supplemental methods

MRI protocol

Assessment form

Supplemental tables

Table I. Histopathological characteristics of identified lesions in sporadic CAA and Dutch-type hereditary CAA

Supplemental figures

Figure I: Histopathological characteristics of CMIs and CMBs in lobar and non-lobar ICH patients

Figure II: Exploratory comparison between sporadic CAA and D-CAA

Supplemental references

**Supplemental methods**

*MRI protocol*

The protocol included a 3D fluid attenuated inversion recovery (FLAIR) sequence (acquired isotropic resolution of 400×400×400 μm^3^, repetition time (TR) = 8,000 ms, nominal echo time (TE) = 164 ms, scan duration 5 hours 20 minutes 8 seconds), a 3D T_2_-weighted turbo spin echo (acquired isotropic resolution of 400×400×400 μm^3^, TR = 3,500 ms, nominal TE = 164 ms, scan duration 1 hour 52 minutes 3 seconds), a 3D T_1_-weighted sequence (acquired isotropic resolution of 400×400×400 μm^3^, TR = 7.7 ms, TE = 3.5 ms, TR between inversion pulses 2,000 ms, flip angle 6°, scan duration 1 hour 9 minutes 36 seconds) and a 3D T_2_*-weighted sequence (acquired isotropic resolution of 180×180×180 μm^3^, flip angle 25°, TR = 75 ms, TE = 20 ms, scan duration 4 hours 59 minutes 31 seconds).

**Assessment form**

| **Case** | **Lesion no.** | **Lobe** | **Type of lesion** | **Location lesion in cortex** | **Related vessel identified** | **CAA score (0-3)** | **Capillary CAA (y/n)** | **Vessel wall splitting (0-3)** | **Microaneurysm  (y/n)** | **Fibrinoid  necrosis (y/n)** | **Loss of SMCs  (0-3)** | **Fibrin vessel  wall (0-3)** | **Fibrin surrounding  cells (0-3)** |
| --- | --- | --- | --- | --- | --- | --- | --- | --- | --- | --- | --- | --- | --- |
|  |  |  |  |  |  |  |  |  |  |  |  |  |  |
|  |  |  |  |  |  |  |  |  |  |  |  |  |  |
|  |  |  |  |  |  |  |  |  |  |  |  |  |  |
|  |  |  |  |  |  |  |  |  |  |  |  |  |  |

Location in cortex:

1. Along penetrating cortical arteriole / under pial surface (layers I-III)
2. Deeper in cortex (layers IV-VI)
3. Subcortical

| **Vonsattel and Greenberg score for CAA severity**[1–3] | |
| --- | --- |
| 0: no | No amyloid deposition |
| 1: mild | Amyloid is restricted to the tunica media without significant destruction of smooth muscle cells. |
| 2: moderate | Tunica media completely replaced by amyloid and the vessel is thicker than normal. |
| 3: severe | Extensive amyloid deposition with focal vessel wall fragmentation, double barreling of the vessel wall, microaneurysm formation and leakage of blood through the blood vessel wall |

| **Deramecourt score for severity of arteriolosclerosis, vessel wall splitting and loss of smooth muscle cells**[4] | |
| --- | --- |
| 0: no | Normal aspect |
| 1: mild | Mild thickening of the vessel media |
| 2: moderate | Partial loss of smooth muscle cells in the media, deposits of fibrohyaline material, endothelial proliferation |
| 3: severe | Complete loss of smooth muscle cells in the media with concentric deposits of fibrohyaline material (lipohyalinosis) and lumen stenosis, or fibrinoid necrosis, or micro atheroma, or microaneurysm |

| **Presence of Fibrin in vessel wall and Fibrin in surrounding cells**[5] | |
| --- | --- |
| 0: None | No fibrin present in vessel walls and/or cells surrounding CMI or CMB |
| 1: Mild | Scant fibrin present in vessel walls and/or cells surrounding CMI or CMB |
| 2: Moderate | Some fibrin present in vessel walls and/or cells surrounding CMI or CMB |
| 3: Severe | Widespread fibrin present in vessel walls and/or cells surrounding CMI or CMB |

CAA = cerebral amyloid angiopathy, CMB = cerebral microbleed, CMI = cerebral microinfarct, SMCs = smooth muscle cells

**Supplemental tables**


**Table I. Histopathological characteristics of identified lesions in sporadic CAA and Dutch-type hereditary CAA**

| **Sporadic CAA vs D-CAA** | **Sporadic CAA**^*^  **(n=21 lesions)** | **D-CAA**†  **(n=22 lesions)** | **p-value** |
| --- | --- | --- | --- |
| **Type of lesion**‡ |  |  |  |
| - CMI, n (%) | 7 (33.3) | 12 (54.5) |  |
| - CMB, n (%) | 14 (66.7) | 10 (45.5) |  |
| **Location of lesion** |  |  |  |
| - Occipital, n (%) | 3 (14.3) | 13 (59.1) |  |
| - Parieto-temporal, n (%) | 12 (57.1) | 5 (22.7) |  |
| - Frontal, n (%) | 6 (28.6) | 4 (18.2) |  |
| **Location in the cortex** |  |  |  |
| - Superficial cortex (layers I-III) | 13 (61.9) | 17 (77.3) | 0.33 |
| - Deep cortex (layers IV-VI) | 4 (19) | 4 (18.2) | 0.94 |
| - Subcortical | 4 (19) | 1 (4.5) | 0.19 |
| **Presence of microaneurysms, n (%)** | 0 (0) | 1 (4.5) | 0.32 |
| **Presence of fibrinoid necrosis, n (%)** | 7 (33.3) | 3 (13.6) | 0.16 |
| **CAA score ≥2** | 19 (90.5) | 22 (100) | 0.23 |
| **VWS score ≥2** | 15 (71.4) | 13 (59.1) | 0.53 |
| **Loss of SMCs score ≥2** | 13 (61.9) | 17 (77.3) | 0.27 |
| - - Not available | 2 (9.5) | 2 (9.1) |  |
| **Fibrin leakage score ≥2** |  |  |  |
| - Vessel walls § | 4/8 (50) | 11/20 (55) | 0.81 |
| - Surrounding cells \| \| | 9/17 (52.9) | 11/22 (50) | 0.86 |

CAA = cerebral amyloid angiopathy, CMB = cerebral microbleed, CMI = cerebral microinfarct, D-CAA = Dutch-type hereditary CAA, ICH = intracerebral hemorrhage, SMC = smooth muscle cells, VWS = vessel wall splitting

^*^ Cases: no.2 (2 CMIs, 2 CMBs), no. 7 (4 CMBs), no. 8 (1 CMI, 8 CMBs), no. 9(4 CMIs)

† Case: no 1. (12 CMIs, 10 CMBs)

‡ We performed no statistical test for this variable, because it is a selection of lesions based on MRI

§ In 11 lesions (9 in sporadic CAA patients and 2 in the D-CAA patient) no surrounding (presumably) involved vessels were identified and in 4 lesions (all in sporadic CAA patients) the fibrin(ogen) stained sections were not available

| | In 4 lesions (all sporadic CAA patients) fibrin(ogen) stained sections were not available

**Supplemental figures**

**Figure I. Histopathological characteristics of CMIs and CMBs in lobar and non-lobar ICH cases**

**
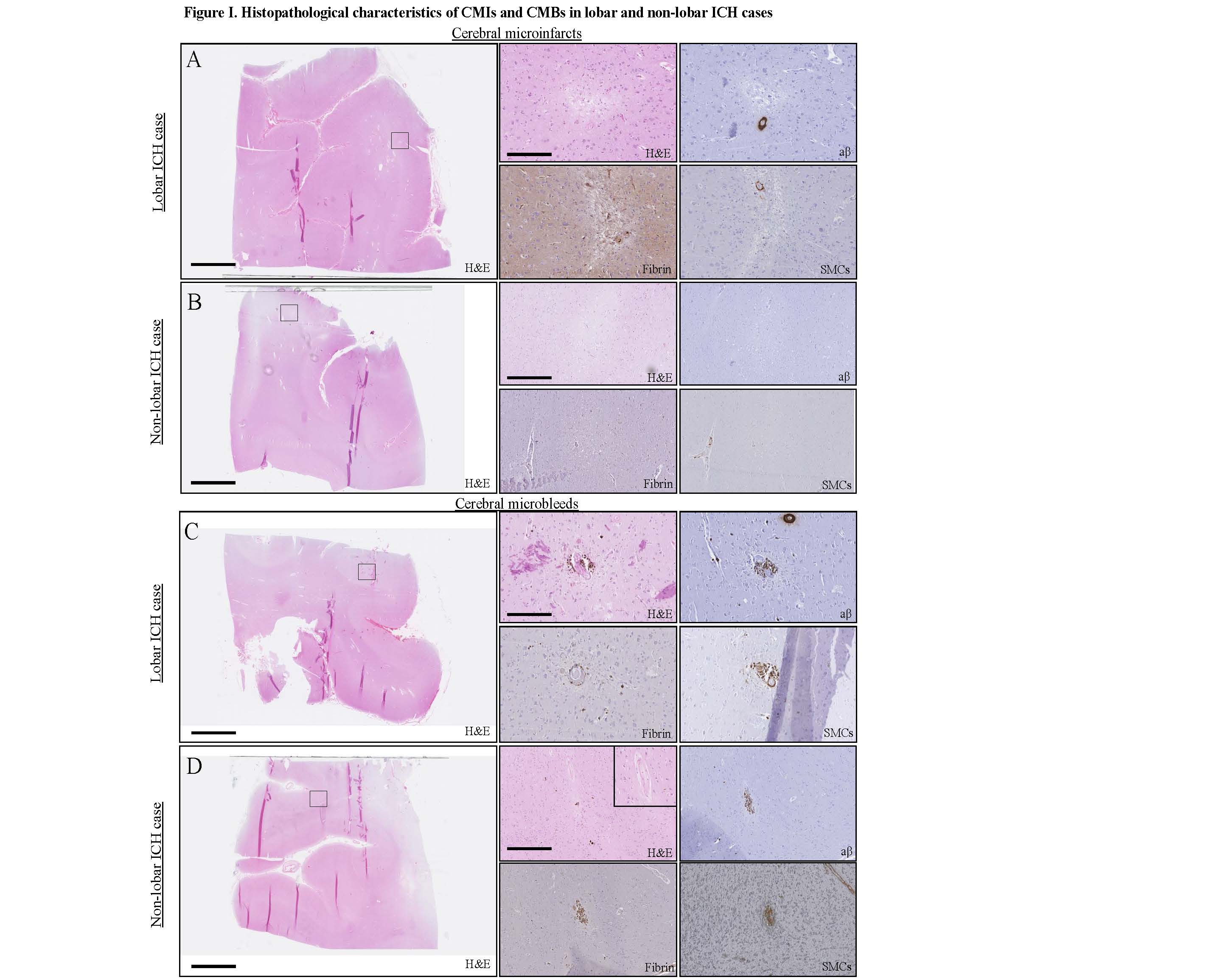
**

Panel A: a chronic CMI in a patient with D-CAA (case no 1.), located in the deeper layers of the cortex, visible as an area with cell loss and gliosis on H&E, a severe CAA score of the section, a moderate vessel wall splitting score, severe uptake of fibrin in the surrounding cells, and moderate loss of SMCs of the vessels in the section.

Panel B: an acute CMI in a patient with a deep ICH due to arteriolosclerotic vasculopathy on histopathology (case no. 13), located in the deeper layers of the cortex, visible as tissue pallor and red neurons on H&E, no presence of CAA, a moderate vessel wall splitting score in the section, mild uptake of fibrin in the surrounding vessel walls and a moderate uptake of fibrin in the surrounding cells, and mild loss of SMCs of the vessels in the section.

Panel C: a chronic CMB in a patient with D-CAA (case no. 1) located in the deeper layers of the cortex visible as hemosiderin deposits on H&E, a moderate CAA score of the section but no Aβ in the direct vicinity of the CMB, absence of vessel wall splitting on the section, mild uptake of fibrin in the vessel walls but moderate uptake in the surrounding cells, and mild loss of SMCs in the surrounding vessels and the rest of the section.

Panel D: a chronic CMB in a patient with a deep ICH due to arteriolosclerotic vasculopathy on histopathology (case no. 11), located in the deeper layers of the cortex, visible as hemosiderin deposits on H&E, a mild CAA score of the section, a severe vessel wall splitting score in the section, no uptake of fibrin in the vessel walls and moderate uptake of fibrin in the surrounding cells, and moderate loss of SMCs of the vessels in the section. The inset shows the changes in the involved vessel with mild vessel wall splitting.

Scale bars in the section overviews are 5 mm. The scale bars in the stained sections zoomed in on lesions are 200 µm for lesions A and C and 400 µm for B and D.

**Figure II. Exploratory comparison between sporadic CAA and D-CAA
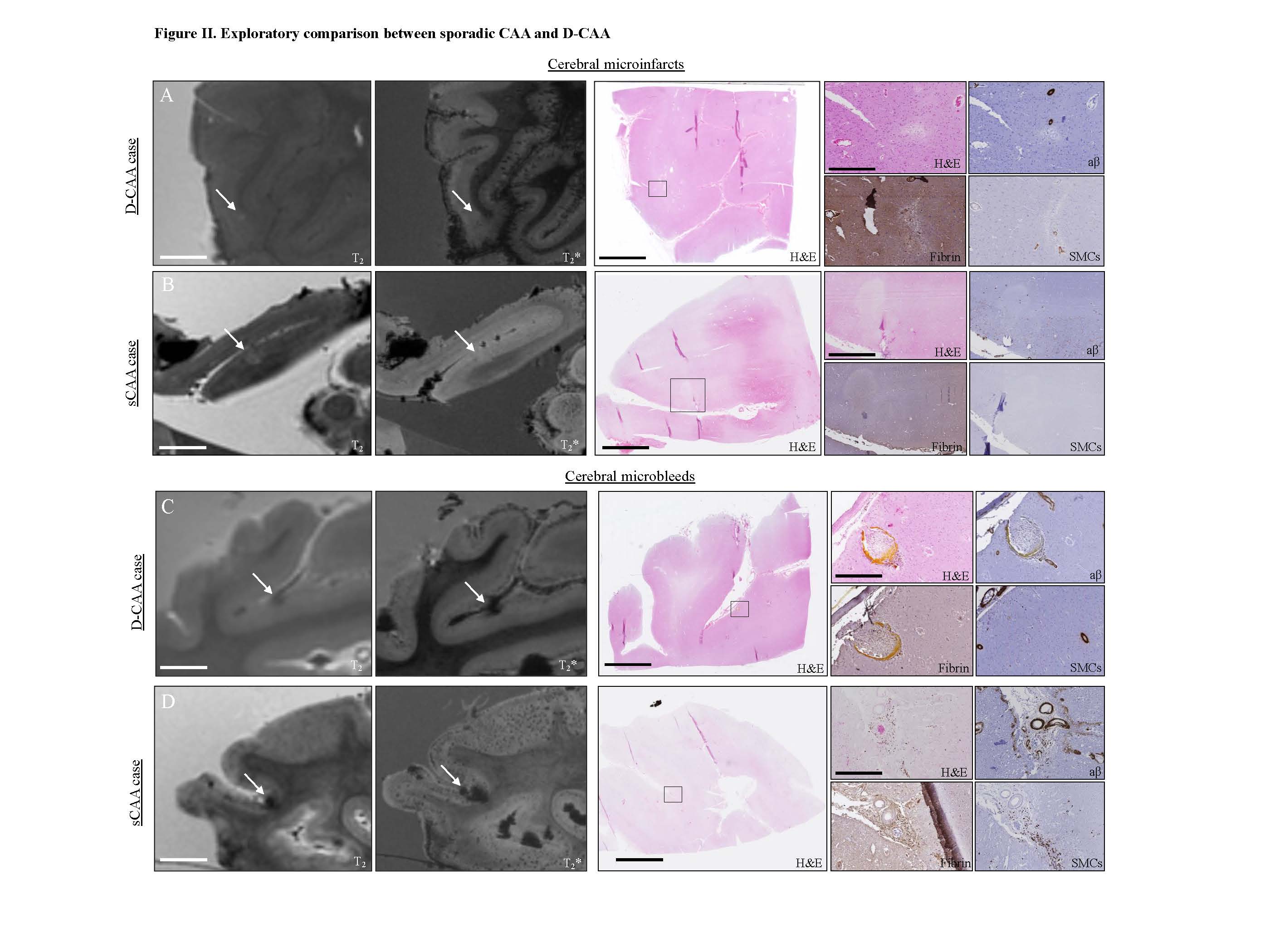
**

Panel A: a chronic CMI in a patient with D-CAA (case no. 1) on ex vivo 7 T MRI T_2_-weighted and T_2_*-weighted images visible as an old CMI on histopathology in the deeper layers of the cortex, visible as cell loss and cavitation on H&E, a severe CAA score on the aβ stain, a moderate vessel wall splitting score in the section, moderate uptake of fibrin in the vessel walls and severe uptake of fibrin in the surrounding cells, and moderate loss of SMCs of the vessels in the section.

Panel B: a CMI in a patient with sporadic CAA (case no. 17) on ex vivo 7 T MRI T_2_-weighted and T_2_*-weighted images visible as an acute CMI on histopathology in the deeper layers of the cortex, visible as tissue pallor and presence of red neurons on H&E, a moderate CAA score on the aβ stain, no presence of vessel wall splitting in the section, mild uptake of fibrin in the vessel walls and no uptake of fibrin in the surrounding cells, and severe loss of SMCs of the vessels in the section

Panel C: a CMB in a patient with D-CAA (case no. 1) on ex vivo 7 T MRI T_2_-weighted and T_2_*-weighted images visible as an old CMB on histopathology in the superficial layers of the cortex, visible as hemosiderin and hematoidin deposit on H&E, a severe CAA score on the amyloid stain, a moderate vessel wall splitting score in the section, mild uptake of fibrin in the vessel walls and no uptake of fibrin in the surrounding cells, and moderate loss of SMCs of the vessels in the section.

Panel D; CMB in a patient with sporadic CAA (case no. 7) on ex vivo 7 T MRI T_2_-weighted and T_2_*-weighted images visible as an old CMB on histopathology in the superficial layers of the cortex, visible as hemosiderin deposits on H&E, a severe CAA score on the amyloid stain, a mild vessel wall splitting score in the section, mild uptake of fibrin in the vessel walls and no uptake of fibrin in the surrounding cells, and severe loss of SMCs of the vessels in the section.

Scale bars in the T_2_ and T_2_* weighted images are 10 mm; in the H&E section overviews 5 mm, in the stained sections zoomed in on lesions are 400 µm for lesions panels A, C and D and 2,5 mm for panel B.

**Supplemental references**

1. Greenberg SM, Vonsattel JPG. Diagnosis of cerebral amyloid angiopathy: Sensitivity and specificity of cortical biopsy. Stroke. 1997;28:1418–22.

2. Poyuran R, Mahadevan A, Arimappamagan A, Nandeesh BN, Nagappa M, Saini J, et al. Cerebrovascular pathology in cerebral amyloid angiopathy presenting as intracerebral haemorrhage. Virchows Archiv. Virchows Archiv; 2019;474:235–45.

3. Vonsattel JPG, Myers RH, Tessa Hedley‐Whyte E, Ropper AH, Bird ED, Richardson EP. Cerebral amyloid angiopathy without and with cerebral hemorrhages: A comparative histological study. Annals of Neurology. 1991;30:637–49.

4. Deramecourt V, Slade JY, Oakley AE, Perry RH, Ince PG, Maurage CA, et al. Staging and natural history of cerebrovascular pathology in dementia. Neurology. 2012;78:1043–50.

5. Freeze WM, Bacskai BJ, Frosch MP, Jacobs HIL, Backes WH, Greenberg SM, et al. Blood-Brain Barrier Leakage and Microvascular Lesions in Cerebral Amyloid Angiopathy. Stroke. 2019;50:328–35.
